# Soil Erosion as a Driver of Eutrophication: An Analysis of European Lakes Using Sentinel‐2 Satellite Data

**DOI:** 10.1111/gcb.70494

**Published:** 2025-09-12

**Authors:** Surya Gupta, Simon Scheper, Pasquale Borrelli, Panos Panagos, Christine Alewell

**Affiliations:** ^1^ Department of Environmental Sciences University of Basel Basel Switzerland; ^2^ Dr. Simon Scheper—Research | Consulting | Teaching Dähre Germany; ^3^ Department of Science Roma Tre University Roma Italy; ^4^ European Commission Joint Research Centre (JRC) Ispra Italy

**Keywords:** bloom occurrence, floating algae index, lakes, maximum bloom extent, nutrients, Sentinel 2

## Abstract

Soil erosion by water is a critical factor contributing to eutrophication in water bodies, acting as a significant source of nitrogen and phosphorus from land. Many models predict soil erosion and sediment transport into lakes and rivers, and the connection between soil erosion triggering eutrophication is considered textbook knowledge. However, limited data‐based scientific evidence exists on the consequences of soil erosion and sediment fluxes on eutrophication. This study examines the impact of soil erosion on eutrophication, considering other covariates such as slope, elevation, phosphorus, nitrogen, flow accumulation and temperature, by analyzing zones of varying sizes around lakes in six different countries of Europe covering an area of 1596 km^2^: Austria (81 lakes), France (310), Germany (266), Hungary (73), Poland (465), and the United Kingdom (316). We utilized multispectral Sentinel‐2 satellite remote sensing data at 20‐m spatial resolution for 2021 and 2022 to estimate the Floating Algae Index (FAI) of lakes. FAI allowed us to quantify bloom occurrence (BO)—the frequency of detected algal blooms—and maximum bloom extent (MBE)—the total area affected by blooms during the study period. The MBEs were then correlated with the aforementioned covariates within zones of 100 m, 200 m, 500 m, and 1 km distance from the lakes using machine learning algorithms to identify the most significant and thus driving factors within these areas. Our results prove quantitatively that soil erosion is indeed a key driver of eutrophication for all the selected European regions except Austria. Water temperature, nutrient input, and slope are additional important drivers of lake eutrophication.

## Graphical abstract

This is one of the first data‐driven studies providing direct evidence that soil erosion drives lake eutrophication on larger scales. By analyzing algal blooms in over 1500 lakes across six European countries (a: United Kingdom, b: Germany, c: France, d: Poland, e: Austria, and f: Hungary) using satellite data and machine learning, soil erosion scored as the most important contributor, in conjunction with factors such as temperature, nutrients, and slope. These findings provide quantitative direct evidence linking soil erosion to declining water quality.

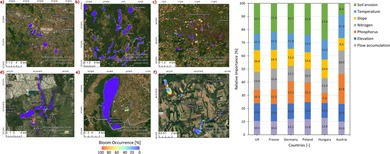

## 1 Introduction

Harmful algal blooms (HABs) have become an increasingly significant threat to both freshwater and marine environments, raising serious concerns for ecosystem health and public safety worldwide (Dai et al. 2023; Hou et al. 2022). In the last 40 years, such blooms have been observed in over 20,000 lakes across the globe, collectively impacting around 57% of the global lake surface area (Hou et al. 2022). Recent studies have shown that 620 out of 1956 large freshwater lakes have experienced algal blooms in over half of the past 20 years, with 504 lakes showing a clear upward trend in bloom frequency, especially after 2015 likely linked to climate warming (Wang et al. 2025). This alarming rise in bloom events reflects not only climate‐related drivers but also intensified land use management with increases in nutrient loading from surrounding catchments (Beusen et al. 2022).

A key contributor to this nutrient enrichment is soil erosion, an environmental process that not only depletes soil fertility, reduces water retention capacity, and lowers crop productivity but also has far‐reaching off‐site impacts, particularly on aquatic ecosystems (Owens 2020; Quinton and Fiener 2024). Among the most serious consequences of soil erosion is eutrophication, as phosphorus (P) and nitrogen (N) losses from agricultural landscapes are major drivers of HAB formation in lakes and rivers (Carpenter 2005; Ge et al. 2023). Nitrogen is primarily lost to streams and drinking water reservoirs through leaching, contributing to eutrophication, acidification, and drinking water contamination. In contrast, phosphorus is largely insoluble in soils and is predominantly lost through soil erosion rather than leaching. Most soil P is tightly bound to mineral particles or incorporated into organic matter, making it unavailable for transport in its dissolved form (Carpenter and Bennett 2011; Alewell et al. 2020). Only a small fraction of soil and bedrock P is plant available or leachable as dissolved phosphate (Helfenstein et al. 2018).

Modeling studies conclude that soil erosion plays a significant role in phosphorus transport, with sediment delivery ratios varying widely across watersheds (Alewell et al. 2020). As such, erosion‐based phosphorus input might rival or even exceed sewage‐derived sources, intensifying eutrophication risks (Krasa et al. 2019). A recent study indicates that soil erosion by water is a major driver of phosphorus (P) loss within the agricultural management chain, with agricultural soils worldwide losing 4–19 kg ha^−1^ yr^−1^ due to soil erosion, equivalent to over 50% of total phosphorus losses within the agricultural system (Alewell et al. 2020). Results from the European Union estimate phosphorus losses from agricultural land due to water erosion at approximately 2 kg ha^−1^ yr^−1^, though with considerable variability across countries (Panagos et al. 2022; Muntwyler et al. 2024). However, both the global and the European study do not address the direct link between soil erosion, phosphorus loss, and eutrophication of adjacent waters.

Considering soil erosion or phosphorus concentration as covariates to assess their impact on water quality of lakes or rivers has been suggested before. For example, Lin et al. (2016) investigated the contribution of soil erosion to lake eutrophication by examining the relationship between soil erosion and algal coverage (a proxy for lake water quality) in three eutrophic lakes in China. They analyzed this relationship at varying distances from the lakes (1, 10, 20, and 40 km). Their findings suggest that soil erosion can serve as a useful indicator for identifying key watershed protection areas. The selection of relevant covariates and the variations of significance across distance for assessing water quality has been explored in various studies (Su et al. 2015). Chang (2008) analyzing eight covariates to determine their influence on water quality yielded better correlations within a close area around the lake (100 m) than across the entire catchment. Szpakowska et al. (2022) further demonstrated that land use, particularly agriculture within a 100‐m buffer, had a greater impact on small water bodies than land use across the entire catchment, emphasizing the importance of spatial scale in water quality assessments. However, none of these studies included soil erosion as a driver of water quality deterioration or eutrophication.

The assessment of eutrophication on a large scale has become more accessible with the availability of high‐resolution remote sensing data. Over the past decade, numerous studies have utilized freely available satellite data, such as Sentinel and Landsat, to map eutrophic areas (Liu et al. 2021; Luo et al. 2023). In recent years, some studies have advanced this approach by integrating multiple technologies, including satellite data, UAV (drone) monitoring, and in situ measurements to enhance the monitoring, simulation, and early warning of HABs (Qiu et al. 2025). The use of remote sensing data has enabled the calculation of indices like the Normalized Difference Vegetation Index (NDVI) and Floating Algae Index (FAI), which capture eutrophication patterns based on spectral reflectance (Visitacion et al. 2019). Numerous studies have already utilized FAI to detect the presence of algal blooms on water surfaces (Lin et al. 2016; Muzhoffar et al. 2023). Hou et al. (2022) conducted a global assessment of eutrophication from the 1980s to the 2010s, producing maps of bloom occurrence (BO)—the frequency of detected algal blooms—and maximum bloom extent (MBE)—the total affected area—at a 1° × 1° spatial resolution. However, these eutrophic indicators were not linked to soil erosion.

Here, we aim to assess the role of soil erosion on eutrophication in European lakes using multispectral satellite imagery (Sentinel 2) from 2021 to 2022, identifying spatial changes and the difference between the 2 years. We evaluate how the importance of different environmental covariates varies with buffer sizes of 100, 200, 500, and 1000 m around the lakes with the hypothesis that soil erosion is the key confounding factor of eutrophication. We also aim to determine how the influence of soil erosion changes with distance from the affected water bodies. By integrating high‐resolution remote sensing data with spatial analysis, this study assesses the direct link between soil erosion and eutrophication of adjacent waters.

## 2 Methodology

### 2.1 Site Selection and Remote Sensing Data

**FIGURE 1:**
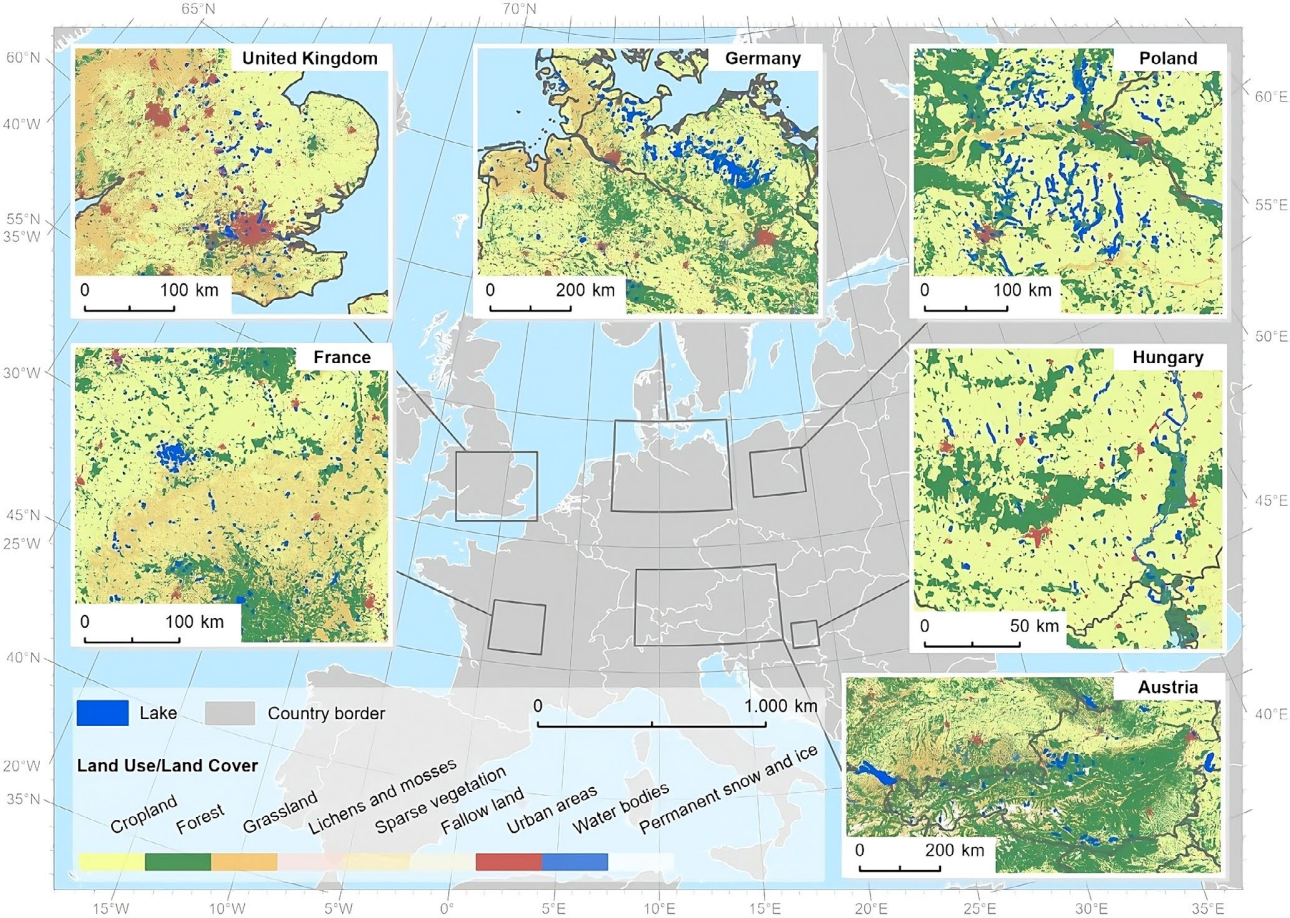
Selected regions (outlined by gray bounding boxes) and associated lakes used to assess the relationship between eutrophication areas and soil erosion. The regions include: (i) United Kingdom, (ii) France, (iii) Germany, (iv) Austria, (v) Poland, and (vi) Hungary. The background layer shows land use and land cover data for the year 2020, obtained from the European Space Agency (ESA) Climate Change Initiative (Land Cover CCI 2017). Map lines delineate study areas and do not necessarily depict accepted national boundaries.

The sites were selected from Central Europe (Germany, Austria, Hungary, and Poland) and North‐western Europe (United Kingdom and France, Figure 1). We focused on regions with a high density of lakes, prioritizing the areas with intense agricultural practices where soil erosion and fertilizer runoff can be expected to be a key driver of eutrophication. We have selected it based on Google Earth Pro by overlying the lake's shapefile. Note that the reference to country names only indicates the evaluated subset of lakes included in this study. We focused on lakes in this study because they often have limited water exchange and long residence times, which make them more vulnerable to nutrient accumulation and persistent eutrophic conditions compared to flowing systems like rivers or well‐mixed systems like oceans (Carpenter et al. 1998). However, we acknowledge that rivers can also experience localized eutrophication, particularly near wastewater discharge points or under low‐flow conditions (Schulz et al. 2023). Similarly, coastal regions of oceans may be affected, though open ocean systems tend to be less sensitive due to greater dilution and mixing capacities.

The study utilized Sentinel‐2A and 2B data from 2021 to 2022. The years 2021 and 2022 were selected to test and demonstrate the proposed methodology and assess its outcomes, as the aim of this study was methodological testing rather than long‐term trend analysis. The data were sourced from the Copernicus Data Space Ecosystem (Milcinski et al. 2024), where we accessed the Sentinel Level 2 product, which is already atmospherically corrected and provides surface reflectance.

We focused on images from March to October, as the spring and summer months are more prone to eutrophication compared to winter (Dupuis and Hann 2009). Soil erosion during winter may also contribute to delayed HABs in spring and summer, as nutrient‐rich sediments delivered to lakes during winter can remain available in the water. Rising temperatures and increased sunlight in spring and summer can then promote algal growth. In addition, spring and summer months are the most susceptible to erosion periods in the study area (Ballabio et al. 2017). For each region and year (2021 and 2022), data were downloaded for each month, separated by month, and then mosaicked.

### 2.2 Representation of Algal Area

The areas of algal bloom or eutrophication were estimated using the Floating Algae Index (FAI) (Hu 2009) based on the Sentinel‐2 imagery.

$$\mathrm{FAI}=\left(R_{\mathrm{nir}}-R_{\mathrm{red}}\right)+\frac{\left(R_{\text{swir}}-R_{\mathrm{red}}\right)\times \left(\lambda_{\mathrm{nir}}-\lambda_{\mathrm{red}}\right)}{\left(\lambda_{\text{swir}}-\lambda_{\mathrm{red}}\right)}$$

where 𝑅 is the atmospheric‐corrected reflectance, and 𝜆 is the wavelength (nm). The reflectance corresponds to the near‐infrared (NIR; band 8a), red (band 4), green (band 3), and shortwave infrared (SWIR; band 11) bands, respectively. Sentinel‐2 images with a maximum cloud cover of 1% were selected for this study. The number of images used is detailed in Table S1. The reflectance values were used at a 20‐m resolution since SWIR data is not available at a 10‐m spatial resolution. It was important to define a threshold for FAI to differentiate between algal bloom and non‐algal bloom areas. Several studies, including Visitacion et al. (2019), Auricht et al. (2019), Luo et al. (2023), and Hu et al. (2010), have used a threshold of 0.02 to detect algae on the water's surface. Based on this literature, we adopted a threshold of 0.02 in this study.

#### 2.2.1 Estimation of Bloom Occurrence and Maximum Bloom Extent

**FIGURE 2:**
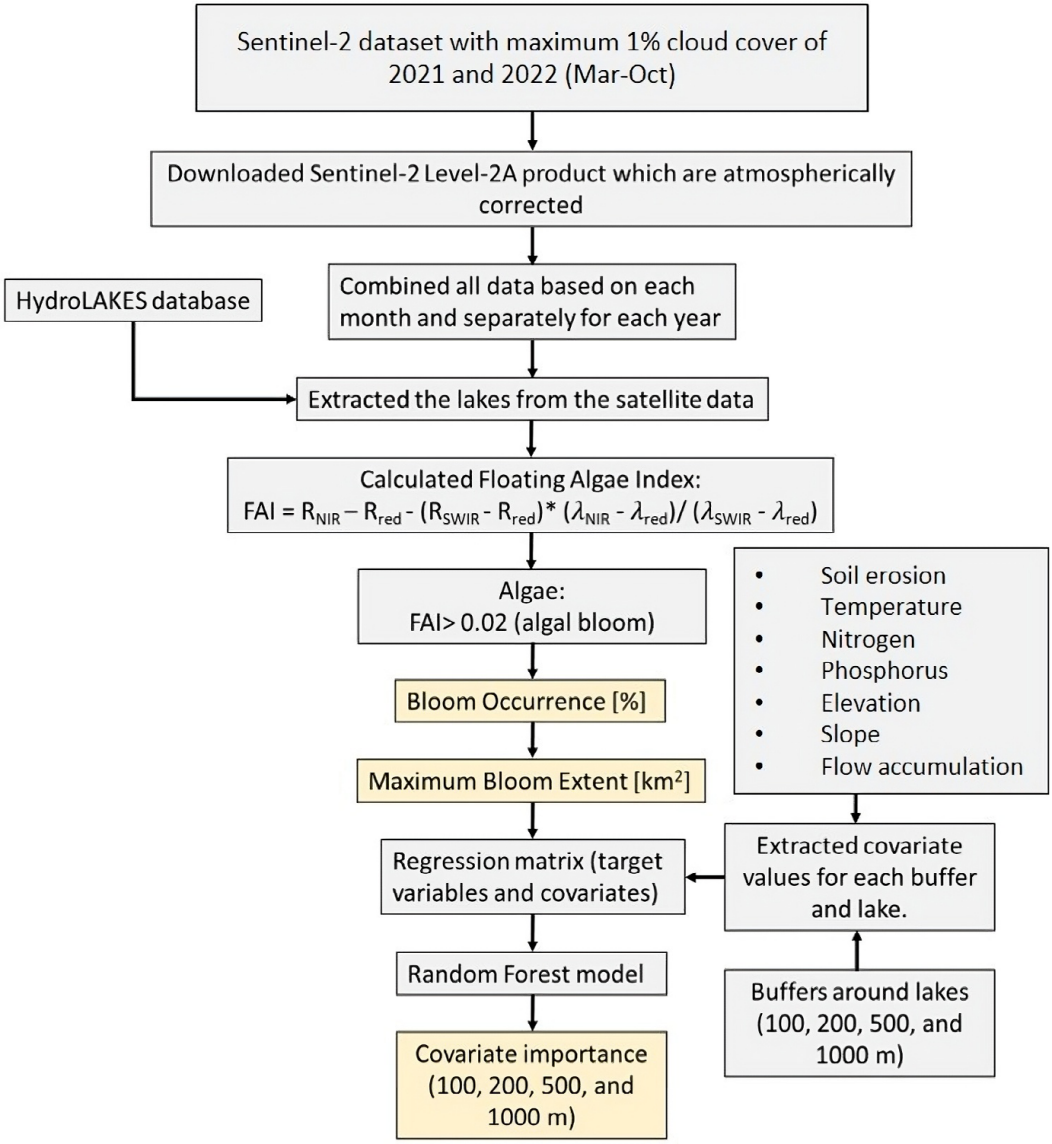
Block diagram describing the methodology to assess crucial driving factors of bloom occurrence and maximum bloom extent in the six investigated regions.

After calculating the FAI, we assessed bloom occurrence and MBE (Figure 2). Bloom occurrence refers to the frequency at which algal blooms were detected during the study period, whereas MBE represents the total area where algal blooms were observed at any point during the study period (Hou et al. 2022), regardless of whether the occurrence happened just once or several times during 2021 and 2022.

We first applied thresholds of 0.02 to the FAI to differentiate between algal bloom and non‐algal bloom areas. For each month in 2021 and 2022, areas identified as algal blooms were assigned a value of 1, whereas non‐algal areas were assigned a value of 0. We then averaged these images using the Cell Statistics tool in ESRI ArcGIS Pro 3.2 by the total number of images to calculate the bloom occurrence percentage over the 2 years. In the resulting sum image, pixels with 0% bloom occurrence were removed, allowing us to sum up the area of each lake affected by algal blooms, which we referred to as the MBE.

### 2.3 Covariates and Buffers

#### 2.3.1 Covariates Selection

The covariates were selected based on factors that likely impact eutrophication. Specifically, we chose temperature (Bouraï et al. 2020), soil phosphorus and nitrogen content of the surrounding buffer zones (Ngatia et al. 2019), elevation, slope, flow accumulation, and soil erosion rate. The temperature data were obtained from the CHELSA dataset (Karger et al. 2017) at a 1‐km spatial resolution. Although this dataset does not directly measure water temperature, it provides the air temperature at 2 m above the surface, as Rollinson and Rowe (2018) found a strong correlation (*R* ^2^ = 0.80) between air and water temperatures. Phosphorus and nitrogen data were obtained from Ballabio et al. (2019) at a 250‐m spatial resolution. Elevation, slope, and flow accumulation data were sourced from the MERIT Hydro dataset (Yamazaki et al. 2019) at a 90‐m spatial resolution. Soil loss rates from water erosion were taken from Borrelli et al. (2018), who applied the WaTEM/SEDEM model to estimate annual erosion rates across Europe at high spatial resolution (25 m).

#### 2.3.2 Buffers Zones

We selected buffer zones around lakes to assess the relationship between eutrophication areas and environmental covariates. Initially, we considered using watersheds and WaTEM/SEDEM output estimate sediment inflow into lakes. Initially, we considered using delineated watersheds along with the output from WaTEM/SEDEM to estimate sediment inflow into lakes. However, accurately delineating watersheds for each lake proved challenging, particularly due to the presence of multiple inlets and complex hydrological connectivity. Many lakes were part of the same watershed according to our analysis and datasets. This limitation made watershed‐based estimation of lake‐specific sediment input impractical. In contrast, using buffer zones allowed us to focus on each lake separately and analyze their contributing factors. We acknowledge that buffer zones are artificial boundaries that may contribute less to eutrophication than naturally built zones of the catchment, where sediments are transported to the lakes directly via streams. Nevertheless, we selected multiple buffer zones at 100, 200, 500, and 1000 m to evaluate how the significance of covariates changes with buffer size and identify key factors driving eutrophication. We limit the distance width to a maximum of 1000 m as many lakes within our study area are in close vicinity and therefore larger buffer zones of lakes would overlap and therefore interfere. Note that we used the mean values of the environmental covariates within each buffer size; however, we used the sum of erosion values as a proxy for sediment.

### 2.4 Fitting Random Forest and Covariate Importance

The MBE, referred to as the eutrophication area, was analyzed in relation to selected covariates using a random forest algorithm to identify the best covariates. The random forest model, implemented in R with the widely used “ranger” package, is effective for prediction in diverse applications (Meloche et al. 2022). The eutrophication area was used as the dependent variable, while the seven potential driving factors were used as independent variables. We fitted the model with default hyperparameters: mtry (set to one‐third of the number of independent variables) and ntree (200) (Zhao et al. 2019). Variable importance was assessed using the residual sum of squares (RSS) metric (Gupta et al. 2021). The covariate with the largest reduction in RSS (indicating lower prediction error) was considered the most significant, and others were ranked by their respective RSS reductions. Note that random forest modeling was employed mainly to assess the relative importance of covariates affecting eutrophication, with the primary objective being variable ranking rather than the construction of a highly predictive model.

## 3 Results

### 3.1 Lake Characteristics and Statistics

This study examines lakes across six European countries: the United Kingdom, France, Germany, Austria, Poland, and Hungary. Among these, the Poland region contains the greatest number of lakes (465), followed by the United Kingdom (316), France (310), and Germany (266). Of the total lakes analyzed, 1247 are classified as small (< 1 km^2^), with Poland accounting for the highest number of small lakes (392). Germany leads in medium‐sized lakes (1–100 km^2^), hosting 131 of the 262 total in this category. Both Germany and Austria each have one large lake (> 100 km^2^). Regarding depth, 812 lakes are shallow (< 3 m), with the United Kingdom having the largest share of shallow lakes (265). Conversely, 699 lakes are categorized as deep (≥ 3 m), most of which are located in Poland (257). Detailed lake statistics by country are summarized in Table 1.

**TABLE 1:** Summary of lake characteristics and bloom metrics across six European countries. The table presents the total number of lakes, categorized into small (< 1 km^2^), medium (1–100 km^2^), and large lakes (> 100 km^2^), along with the average bloom occurrence (BO), percentage, and maximum bloom extent (MBE, km^2^) in 2021 and 2022. The MBE values are further detailed for small‐ and medium‐sized lakes. Note that the size of lakes was taken from Pi et al. (2022).

Regions	UK	Germany	France	Poland	Austria	Hungary
Number of lakes (*N*)	316	266	310	465	81	73
Small lakes (< 1 km^2^)	294	134	300	392	61	66
Medium lakes (1–100 km^2^)	22	131	10	73	19	7
Large lakes (> 100 km^2^)	0	1	0	0	1	0
Shallow lakes (< 3 m)	265	83	204	208	24	28
Deep lakes (≥ 3 m)	51	183	106	257	57	45
Avg. BO lakes 2021 (%)	44	53	57	49	70	39
Avg. BO lakes 2022 (%)	59	47	65	59	71	59
Avg. MBE lakes 2021 (km^2^)	0.130	0.141	0.151	0.139	0.144	0.497
Avg. MBE lakes 2022 (km^2^)	0.139	0.143	0.150	0.102	0.423	0.307
Avg. MBE in smaller lakes 2021 (km^2^)	0.106	0.066	0.131	0.077	0.052	0.291
Avg. MBE in medium lakes 2021 (km^2^)	0.442	0.202	0.770	0.472	0.282	2.439
Avg. MBE in smaller lakes 2022 (km^2^)	0.100	0.070	0.128	0.072	0.102	0.169
Avg. MBE in medium lakes 2022 (km^2^)	0.644	0.212	0.820	0.258	1.475	1.608

### 3.2 Bloom Occurrence and Maximum Bloom Extent

**FIGURE 3:**
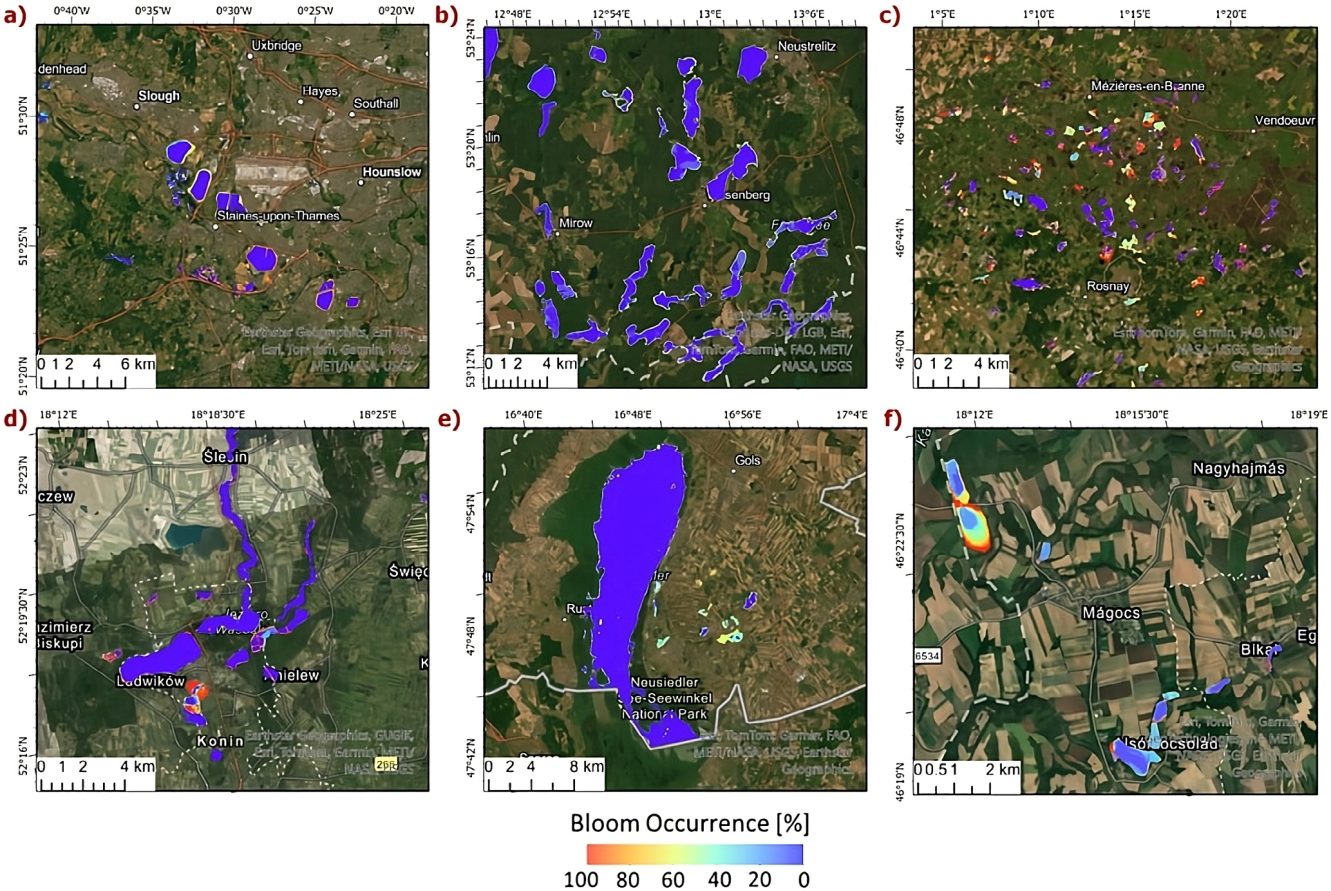
Occurrence of algal blooms in various European region's lakes for combined years (2021 and 2022). (a) United Kingdom, (b) Germany, (c) France, (d) Poland, (e) Austria, and (f) Hungary.

**FIGURE 4:**
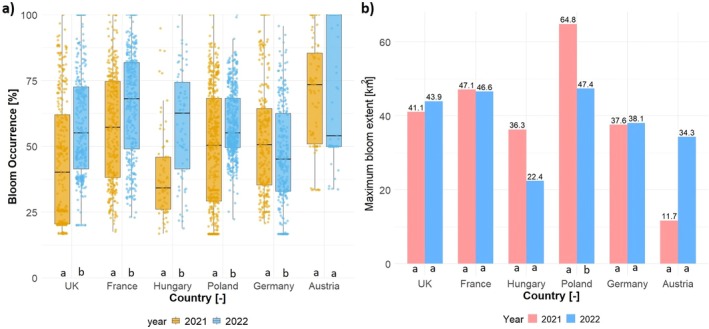
(a) Box plot showing bloom occurrence, and (b) bar plot depicting the maximum bloom extent across different countries. Lowercase letters indicate statistically significant differences between classes (*p* < 0.05), determined by independent *t*‐tests in R.

Examples of bloom occurrence are illustrated in Figure 3, where values near zero (shown in blue) represent clear water or minimal bloom presence, whereas values closer to red indicate areas with high bloom intensity. When combining both years, lakes in Austria showed the highest overall bloom occurrence, followed by those in France, Poland, the United Kingdom, and Germany. Year‐to‐year comparisons revealed that the mean bloom occurrence in 2022 was higher than in 2021 for the United Kingdom, France, Hungary, and Poland, but lower for Germany and Austria (Figure 4a). In all areas except Austria, these differences are statistically significant.

Regarding MBE, the Poland region had the highest MBE in 2021, covering 64.8 km^2^ (22% of the total area), followed by the France region with 47.1 km^2^ (49%) and the UK region with 41.1 km^2^ (30.5%). The total eutrophication area remained nearly the same for most countries in both years, except for the Hungary, Poland, and Austria regions (Figure 4b). Only for the Poland region difference in MBE between 2021 and 2022 was significant. From 2021 to 2022, the eutrophication area decreased by 38.2% in Hungary and 26.8% in Poland, whereas in Austria it increased sharply by 193%. The highest average MBE for both years was observed in Hungary (0.40 km^2^), followed by Austria (0.28 km^2^) and France (0.15 km^2^). When categorized by lake size, Hungary had the highest average MBE for smaller lakes, followed by France and the United Kingdom in both 2021 and 2022. However, Hungary also had the largest eutrophication‐affected areas, averaging 2.4 km^2^ in 2021 and 1.5 km^2^ in 2022 for medium lakes (Table 1).

**FIGURE 5:**
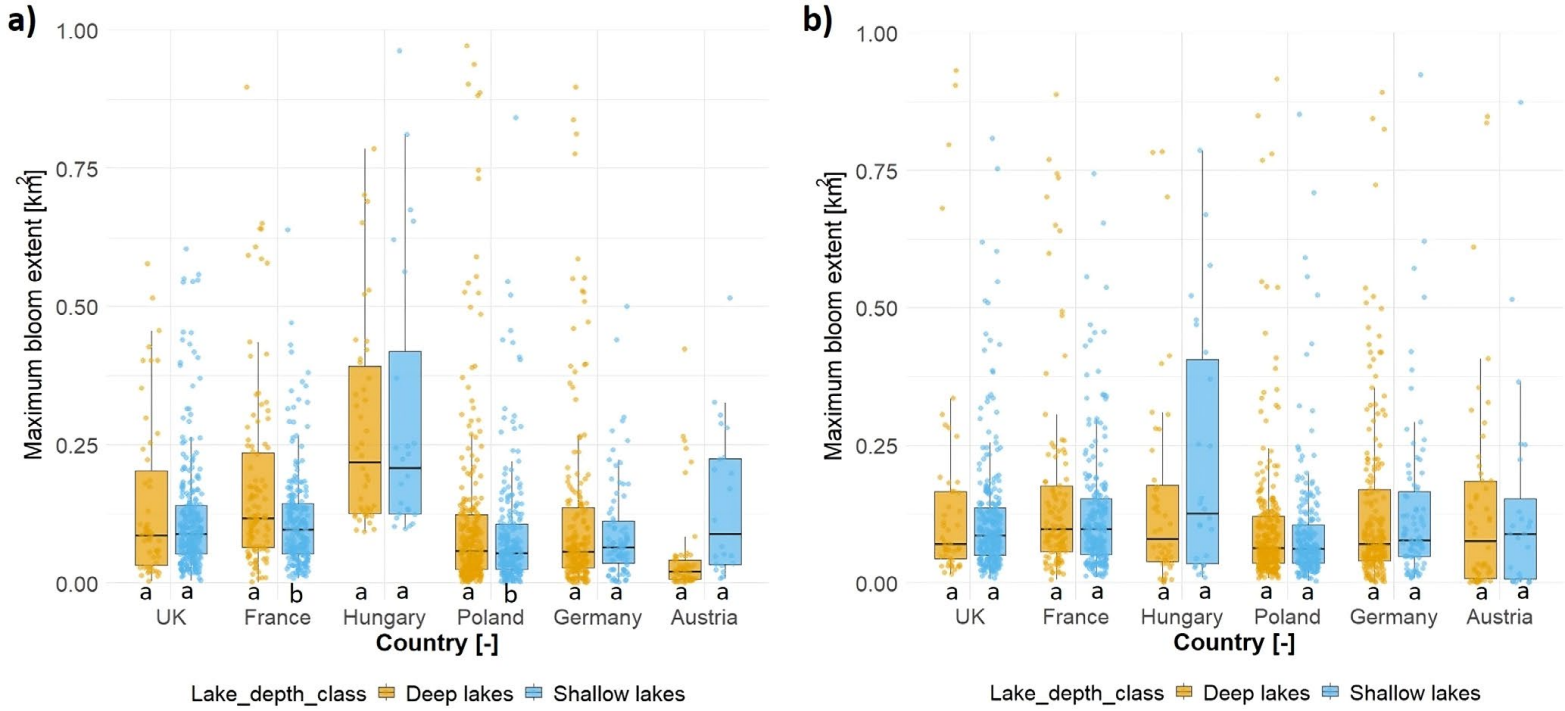
Comparison of maximum bloom extent (MBE) based on lake depth for (a) 2021 and (b) 2022. Shallow lakes are defined as having an average depth of < 3 m, while deep lakes have an average depth of 3 m or more (Poikane et al. 2014). Lowercase letters indicate statistically significant differences between classes (*p* < 0.05), determined by independent *t*‐tests in R.

Regarding the MBE in shallow versus deep lakes, Figure 5a,b indicates that most countries exhibited similar median values, with notable exceptions in France, Hungary, and Austria in 2021, and in the United Kingdom, Hungary, and Austria in 2022. Specifically, in 2021, Austria showed higher median MBE values in shallow lakes, whereas France and Hungary had lower values. In 2022, shallow lakes in Hungary, Austria, and the United Kingdom displayed elevated median MBE values. However, differences between shallow and deep lakes were significant only for France and Poland in 2021, and there were no significant differences for any country in 2022.

### 3.3 Covariate Importance

**FIGURE 6:**
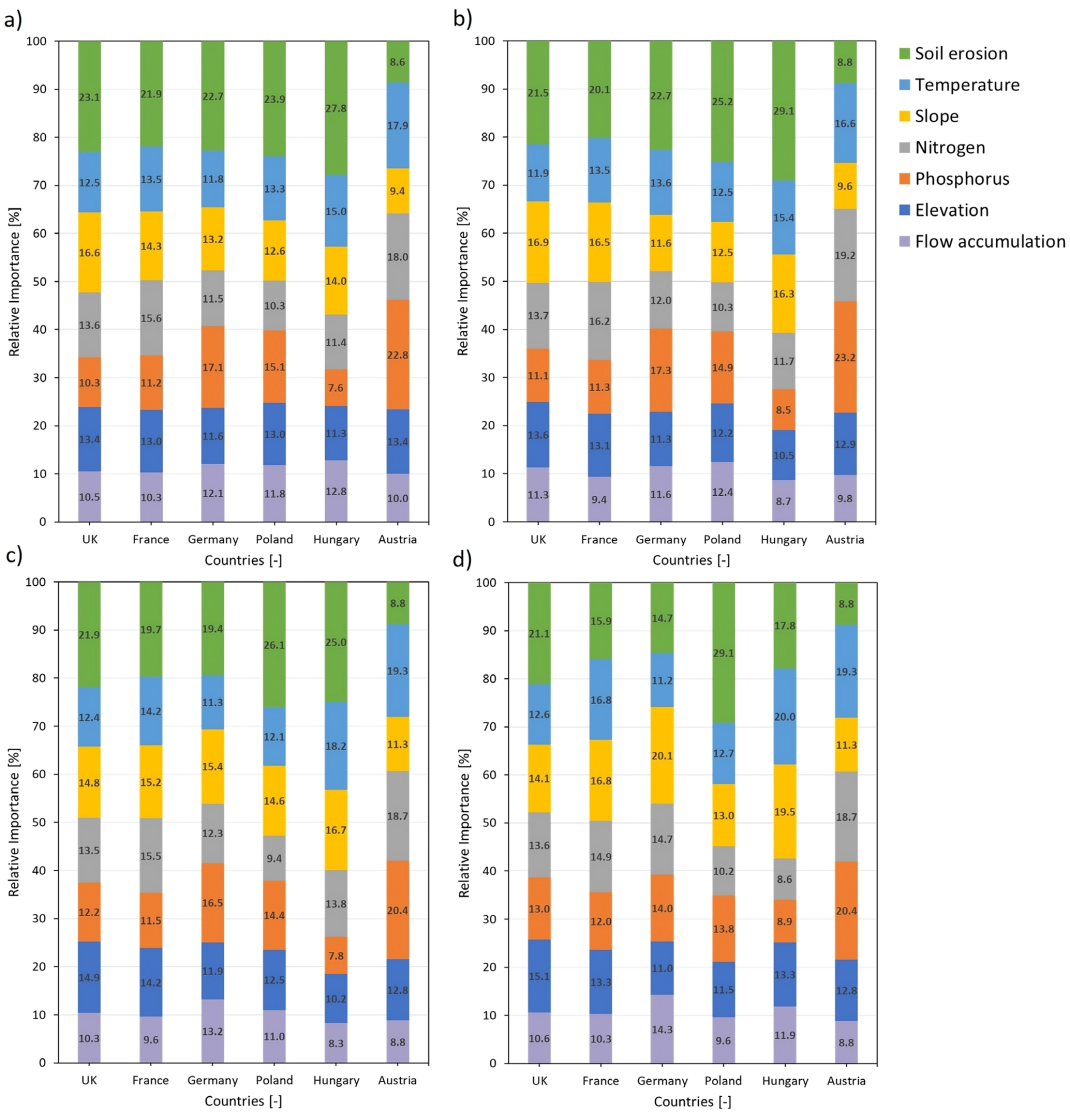
Relative feature importance after applying the FAI threshold of 0.02 across different buffer zones: (a) 100 m, (b) 200 m, (c) 500 m, and (d) 1000 m. The numbers within the plot represent the percentage of relative importance for each covariate.

The covariate importance analysis proved our hypothesis that soil erosion was the most influential factor for all countries except Austria, where phosphorus consistently held the highest importance across all buffers (Figure 6). Phosphorus ranked second in Germany and Poland at the 100 and 200 m buffers. In Hungary, temperature was the second most important covariate, whereas in Poland and Austria it ranked third at 100 m. Nitrogen was the major driver in Austria and France, ranking second across most buffers and placed third in Germany at 100 m but had limited influence elsewhere. Slope showed notable importance in the United Kingdom, ranking second at the two smallest buffers, and was the third most important factor in Germany, France, and Hungary at 100 m. Elevation generally ranked fourth or lower across all countries at the smallest buffer distance. Flow accumulation contributed little in most cases, though its influence was slightly higher in Germany at larger buffers (Figure 6).

**FIGURE 7:**
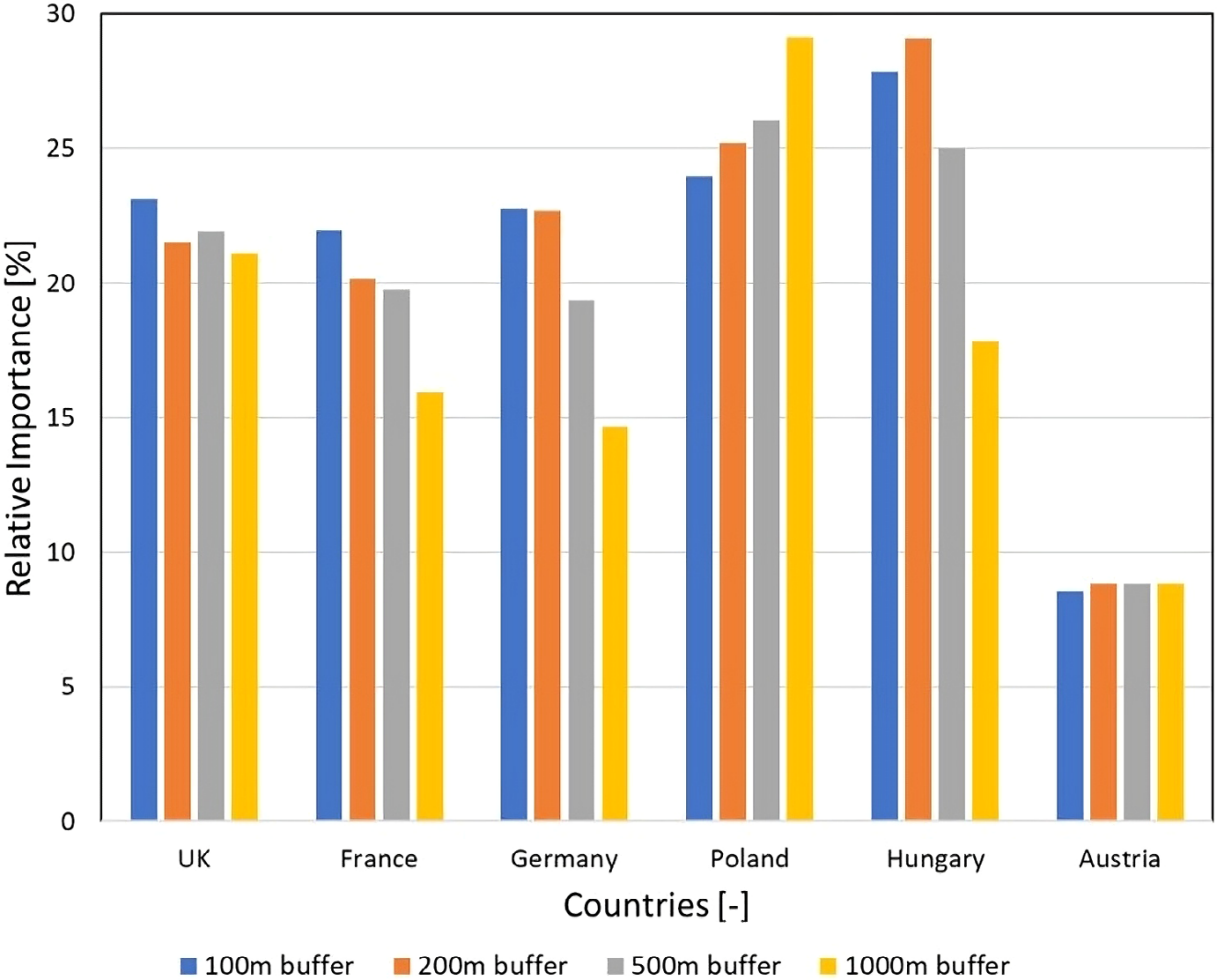
Relative importance of soil erosion covariate.

With increasing buffer distances from 100 to 1000 m, the importance of variables exhibited distinct patterns (Figure 7 and Figure S1). In the lakes of Germany, the significance of soil erosion and phosphorus remained stable at 100 and 200 m buffers and then declined with distance, whereas nitrogen, slope, and flow accumulation gained importance, with slope dipping slightly at 200 m. Elevation and temperature remained stable, except for a small temperature increase at 200 m. A similar decreasing trend for soil erosion was observed in the lakes of France, but rose slightly at 200 m, while temperature and slope significance increased with distance. In contrast, nitrogen, phosphorus, and flow accumulation influence stayed relatively constant, with minor fluctuations such as phosphorus increasing at 1000 m, nitrogen at 200 m, and flow accumulation decreasing at 200 and 500 m. In the lakes of the United Kingdom, soil erosion impact declined with distance but remained constant between 200 and 500 m. Phosphorus and elevation gained importance, while slope decreased, and nitrogen, temperature, and flow accumulation remained stable.

For the lakes of Poland, soil erosion importance increased with distance, whereas phosphorus and elevation decreased. Flow accumulation also declined but rose slightly at 200 m, and other variables remained constant except for a small increase in slope for the 500 m buffer. In the lakes of Hungary, soil erosion influence was low at 100 m, peaked at 200 m, and then decreased. Elevation and flow accumulation importance also declined with distance; however, they rose again at 1000 m, while temperature and slope increased. The influence of phosphorus rose slightly with distance and remained constant for the 100 and 500 m buffers. For the lakes of Austria, soil erosion, nitrogen, and elevation importance were relatively stable across all buffers. Phosphorus and flow accumulation impact decreased with distance, while slope increased, and temperature showed a slight rise with a small dip at 200 m.

## 4 Discussion

### 4.1 Maximum Bloom Extent

The MBE was highest in lakes in Poland in 2021 (64.8 km^2^; Figure 4b). This aligns with previous findings from the Poznań Lakeland, which demonstrated substantial seasonal and interannual variability in nutrient concentrations (particularly nitrates), indicating ongoing eutrophication pressures. Despite some advancements in wastewater management, persistent nutrient inputs from agricultural runoff continue to drive eutrophic to hypertrophic conditions in these lakes (Zbierska et al. 2016). Similarly, lakes in both the United Kingdom and France showed high eutrophication levels in 2022, with MBEs of 43.9 and 46.6 km^2^, respectively. Although nutrient inputs have declined in the United Kingdom over the past four decades, they still exceed safe ecological limits (Environment Agency 2024; Jarvie et al. 2025), which is consistent with our detection of large bloom areas in the British lakes. Similarly, in northern Germany, where our results show high eutrophication, over 70% of lakes still fail to meet “good ecological status” under the European Water Framework Directive (Rücker et al. 2019). This suggests that diffuse nutrient emissions from agriculture, exacerbated by soil erosion, remain a significant driver as our study could demonstrate. Hungary's lakes were also affected by substantial MBE in both years (36.3 km^2^ in 2021, 22.4 km^2^ in 2022). This pattern may relate to recent climatic shifts and changes in lake management, which can trigger internal eutrophication. For example, Istvánovics et al. (2022) documented a severe bloom in Lake Balaton in 2019, despite decades of nutrient reduction measures, highlighting the role of warming‐induced internal nutrient cycling.

Shallow lakes in Germany, Austria, the United Kingdom, and Hungary (in 2022) exhibit slightly higher MBEs compared to deep lakes (Figure 5). This may be attributed to the fact that sunlight penetrates the entire water column in shallow systems, enhancing (i) algal growth within the whole water column, and (ii) resuspension of nutrients from bottom sediments into surface waters (Han et al. 2021). Moreover, the lower water volume in shallow lakes reduces dilution capacity, while intensified human activities, higher external nutrient inputs, and elevated temperatures further exacerbate eutrophication (Zhou et al. 2022). This observation aligns with Poikane et al. (2014), who analyzed 810 lakes across Central Europe and found significantly higher chlorophyll‐*a* concentrations (an indicator of algal blooms) in shallow lakes compared to deeper ones.

### 4.2 Multiple Factors Affect Eutrophication

Although soil erosion emerged as the strongest driver of MBE across most countries, our findings confirm that eutrophication is shaped by a network of interacting factors, including temperature, nutrients, elevation, and slope (Figure 6). For example, while temperature alone may promote floating plant growth rather than blooms (Feuchtmayr et al. 2009), elevated temperatures in nutrient‐rich systems likely exacerbate bloom intensity. Elevation plays an indirect role by affecting phosphorus availability in the soil; in higher altitude areas, phosphorus‐rich topsoil is more susceptible to runoff, increasing the risk of nutrient loading in water bodies (Wu et al. 2024). Slope gradients further enhanced this issue, as steeper slopes (e.g., 20°) accelerate surface runoff and sediment loss, leading to higher phosphorus transport (He et al. 2020). In addition to these terrestrial influences, Figure S2 highlights the critical interplay between phosphorus inflow concentration, water residence time, and lake trophic state. Higher phosphorus inflow concentrations and longer water retention periods promote eutrophic and hypereutrophic conditions, elevating chlorophyll‐*a* levels and algal proliferation. Thus, eutrophication is a multifaceted process driven by a combination of nutrient input, hydrology, and landscape characteristics. Recognizing these interactions is essential for effective lake management and the prevention of excessive nutrient enrichment. Nevertheless, the consistency of soil erosion as the dominant predictor suggests that terrestrial management remains the most direct lever for reducing bloom risk, even as climatic and morphological variables shape local outcomes.

### 4.3 Relationship Between LULC and Maximum Bloom Extent

**FIGURE 8:**
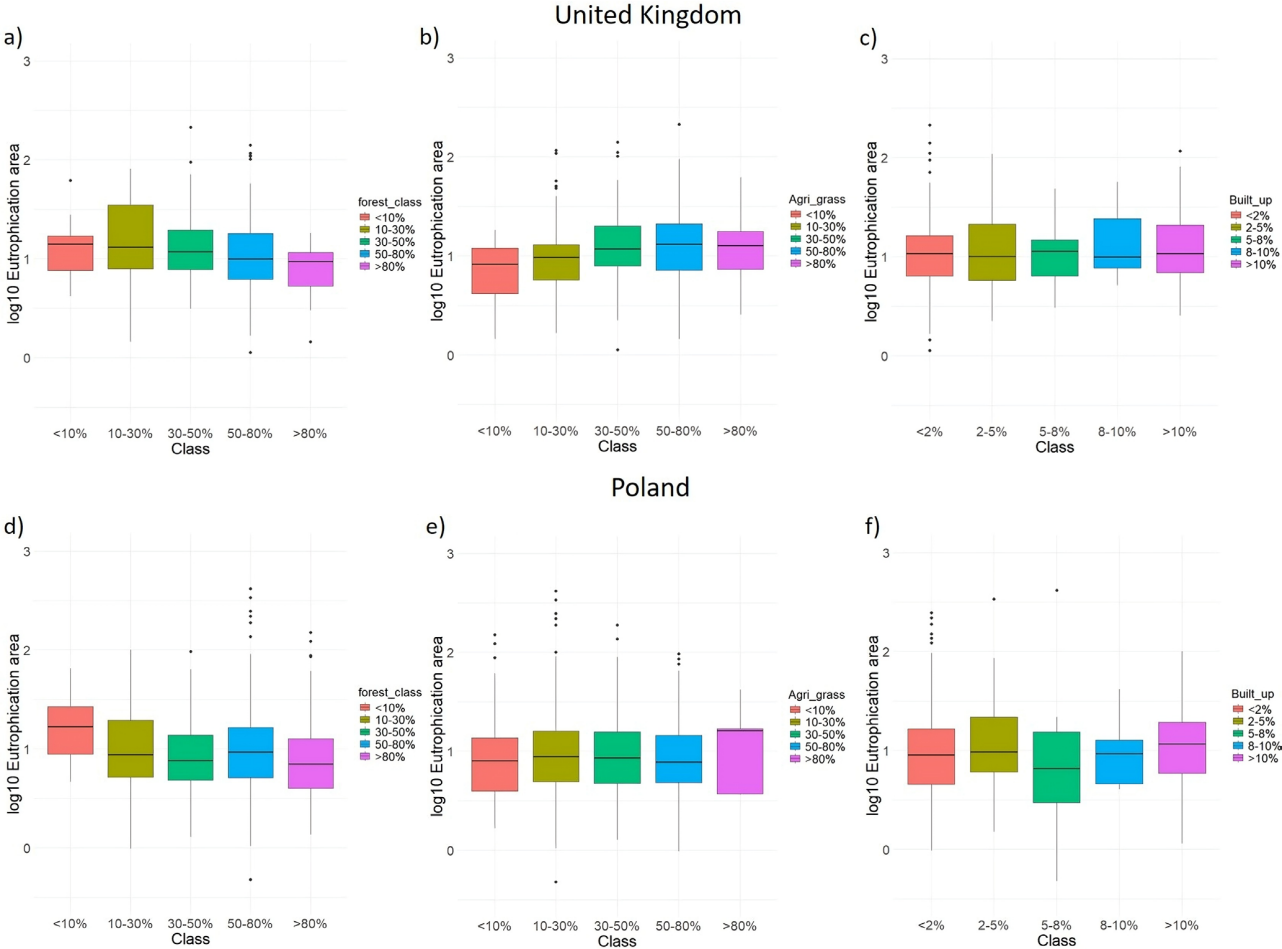
Relationship between eutrophication area/maximum bloom extent and the percentage of different land use/land cover (LULC) types. Panels (a, d) depict forest cover, (b, e) represent agricultural land, and (c, f) illustrate built‐up areas for United Kingdom and Poland, respectively. Note the unit of eutrophication area is log_10_ ha. For Germany, Austria, Hungary, and France lakes see Figure S3.

The relationship between land use/land cover (LULC) and MBE was also examined (see Data S1 for methods). While several studies have reported associations between LULC and the extent of algal blooms (Zhu et al. 2024; Marion et al. 2017), our analysis did not reveal a consistent correlation between MBE and the percentage of forest, agricultural, or built‐up areas (Figure 8). For instance, in the UK and Poland regions, an increase in forest cover within the 100 m buffer zone was associated with a decrease in MBE, whereas MBE tended to increase with a greater percentage of agricultural areas and showed little change with increases in built‐up areas. In contrast, no such clear pattern was observed in the France and Austria regions. In Hungary, MBE decreased as forest cover increased, but no consistent trend was observed for agricultural areas. Interestingly, in the Germany region, MBE actually declined as agricultural area increased (Figure S3), suggesting that the LULC–MBE relationship is highly region specific and complex. These findings are consistent with Lin et al. (2015), who demonstrated that the relationship between agricultural land fragmentation and algal blooms in Lake Taihu varied across different regions. The absence of a uniform trend across regions may be attributed to a combination of factors, including regional environmental variability, internal nutrient loading dynamics, spatial scale, and methodological constraints.

### 4.4 Removing Interdependent Covariates and Assessing Their Impact on Variable Importance

Some covariates used in the model are intercorrelated. For instance, a significant portion of phosphorus enters water bodies via soil erosion, a process strongly influenced by slope while temperature typically decreases with elevation. To assess the impact of such dependencies on variable importance, we conducted two tests: (1) removing phosphorus content as a covariate, and (2) excluding both slope and elevation. These tests aimed to determine whether the exclusion of interdependent covariates would significantly alter the relative importance of the remaining predictors.

When phosphorus was removed as a covariate, the overall pattern of covariate importance remained largely consistent with the model using all seven covariates, although some shifts were observed (Figure S4). Soil erosion remained the most important predictor for the lakes in the United Kingdom, France, Poland, Hungary, and Germany. In Austria, where phosphorus was the most important driver, nitrogen and temperature became the top two predictors across all buffers. For the lakes of Poland, the importance of temperature and elevation increased at all buffer distances. The relationship between temperature and phosphorus was also highlighted by Gianniny et al. (2024), who reported that elevated soil temperatures can enhance phosphorus mobilization from mountain soils, although this effect may be constrained under low soil moisture conditions. Notably, slope showed a marked increase in importance, especially for the lakes in Germany, where it ranked second for all buffers except the 1000 m buffer, where it became the top covariate, mirroring the trend in Figure 6. In Hungary, slope also gained importance with distance, becoming the leading covariate at 1000 m. These shifts suggest that while phosphorus plays an important role, other covariates, particularly slope and temperature, can capture similar environmental signals depending on the country and spatial scale. Numerous studies also support the notion that higher slopes are associated with greater phosphorus loss (Deng et al. 2019; He et al. 2020).

In the second scenario, where both slope and elevation were excluded, soil erosion remained the top covariate for the lakes in the United Kingdom, France, Poland, Hungary, and Germany. Overall patterns were similar to the full model, but the covariates increased in relative contribution (Figure S5). Phosphorus importance rose in Germany, Poland, and Austria across all buffers, while temperature and nitrogen also gained influence in most countries. Temperature ranked second for Hungary and Austria, and third for other countries across all buffers. Nitrogen ranked second for the United Kingdom and France. These results reinforce that although soil erosion and phosphorus are influential, other covariates, especially temperature and nitrogen, can serve as strong proxies depending on the spatial context.

### 4.5 Impact of Different Thresholds

The impact of varying the threshold used to calculate the Floating Algae Index (see above) from 0.02 to 0.05 was also evaluated by assessing changes in MBE. This range was selected based on findings by Garcia et al. (2013), who reported algae pixel values between approximately 0.02 and 0.07, and Liu et al. (2021), who identified a threshold of 0.0693 using bimodal histograms of FAI images for floating algae detection. The most significant reduction in MBE was observed in the lakes of Poland, where the area decreased from 79.5 to 47 km^2^, followed by the lakes in France (from 64.8 to 41 km^2^) and Austria (from 44.2 to 30.4 km^2^) (see Figure S6a). In terms of variable importance at the 100 m buffer, soil erosion remained the most influential predictor for the lakes in the United Kingdom, France, Hungary, Poland, and Germany, consistent with results from the 0.02 threshold (Figure S6b). The importance of temperature and elevation increased in France, slope gained importance in Poland, and nitrogen became more influential in Hungary. In Austria, temperature emerged as the most important covariate at the 0.05 threshold, whereas it ranked third at the 0.02 threshold. This likely occurs because changing the FAI threshold alters the extent and intensity of blooms detected (see Figure S6a). The lower threshold (0.02) captures smaller or less intense blooms influenced by broader environmental conditions, while the higher threshold (0.05) detects only intense blooms linked to more specific factors such as high nutrient loads, steep slopes, or distinct temperature regimes, thereby shifting covariate importance in the model. These differences highlight the importance of careful threshold selection. In this study, the choice of a 0.02 threshold is supported by previous literature (Visitacion et al. 2019; Auricht et al. 2019; Luo et al. 2023; Hu et al. 2010), where it is commonly used. However, other studies, such as Garcia et al. (2013) and Liu et al. (2021), have investigated the use of higher thresholds.

### 4.6 Limitation and Future Work

While conducting this study, we encountered a few limitations that should be considered when interpreting the results. A primary limitation is the use of air temperature as a proxy for water temperature. Although Rollinson and Rowe (2018) reported a strong correlation between the two (*R* ^2^ = 0.80), actual water temperature measurements could potentially strengthen the role of temperature as a covariate influencing eutrophication. However, we could not acquire water temperatures for all the selected lakes (*n* = 1511) for the years 2021 and 2022. Second, buffer zones were used instead of hydrologically defined watersheds to estimate land‐based influences on lakes. While watersheds provide a more relevant spatial unit for modeling sediment inflow, accurate delineation was impractical due to multiple inlets. This limitation highlights the need for high‐resolution DEMs and improved watershed delineation techniques in future research. Third, soil erosion estimates were based on the WaTEM/SEDEM model map produced by Borrelli et al. (2018). Limitations here include simplistic representation of landscape connectivity, reduced prediction accuracy in heterogeneous catchments, sensitivity to calibration catchment selection, and potential overestimation in nonagricultural areas. Therefore, incorporating measured discharge or sediment yield data of respective lake catchments could improve robustness. Fourth, high‐resolution wind speed/direction data were unavailable, even though wind can be an important covariate for algal bloom development. This should be incorporated in future studies. Additionally, the selection of the threshold for the Floating Algae Index (FAI) introduces uncertainty. We used a threshold of 0.02, consistent with most existing literature, but other studies have proposed higher values. As shown in Figure S5, this choice can significantly affect bloom detection, underscoring the need for standardized or data‐driven approaches to threshold selection. Finally, we lacked chlorophyll‐*a* data for validation, which limited our ability to assess the accuracy of algal bloom detection derived from satellite indices. Moreover, while Random Forest modeling effectively ranks the relative importance of covariates, it does not inherently reveal the direction (positive or negative) of their influence on eutrophication, which limits interpretability compared to parametric models.

## 5 Conclusion

This study examines eutrophication patterns in six European regions (United Kingdom, Germany, France, Poland, Austria, and Hungary), focusing on bloom occurrence (BO) and MBE in relation to key environmental drivers. Austria recorded the highest bloom frequency, while Poland had the largest affected areas. From 2021 to 2022, bloom extent increased in Austria but declined in Hungary and Poland, highlighting distinct regional and temporal dynamics of eutrophication.

Random Forest analysis showed that soil erosion was the leading driver of MBE in most countries, except Austria, where phosphorus consistently ranked highest. Nitrogen, temperature, slope, and elevation had country‐specific importance, while flow accumulation played a minor role. Covariate influence often shifted with buffer distance. Soil erosion typically declined with distance in western countries but increased in parts of eastern Europe, while other variables gained or lost importance depending on region. These results highlight strong regional and scale‐dependent variability, indicating that eutrophication is driven by distinct local interactions of nutrients, land use, and topography rather than a single dominant factor.

Our findings suggest that the consistent importance of soil erosion highlights the potential of targeted terrestrial management to reduce eutrophication and bloom risk in lakes, but effective lake management will require strategies tailored to regional conditions and scale‐dependent processes. Future research should incorporate measured sediment yields as covariates and refine threshold‐based detection methods to improve modeling accuracy. This work represents a first step toward statistically quantifying the role of soil erosion in shaping eutrophication patterns across diverse European landscapes.

## Author Contributions

**Surya Gupta:** conceptualization, data curation, formal analysis, methodology, writing – original draft. **Simon Scheper:** conceptualization, data curation, writing – review and editing. **Pasquale Borrelli:** conceptualization, writing – review and editing. **Panos Panagos:** conceptualization, writing – review and editing. **Christine Alewell:** conceptualization, supervision, writing – review and editing.

## Conflicts of Interest

The authors declare no conflicts of interest.

## Supporting information


**Data S1:** Relationship between maximum bloom extent (MBE) and land use land cover.
**Figure S1:** Relative importance of individual covariates: (a) flow accumulation, (b) phosphorus, (c) nitrogen, (d) temperature, (e) elevation, and (f) slope with respect to different buffer sizes.
**Figure S2:** The relationship between phosphorus inflow concentration, water residence time, and trophic state. Figure from Vollenweider and Kerekes (1982).
**Figure S3:** Relationship between eutrophication area/maximum bloom extent and the percentage of different land use/land cover (LULC) types. Panels (a, d, g, j) depict forest cover, (b, e, h, k) represent agricultural land, and (c, f, i, l) illustrate built‐up areas for France, Hungary, Germany, and Austria, respectively. Note the unit of eutrophication area is log_10_ ha.
**Figure S4:** Relative feature importance after applying the FAI threshold of 0.02 across different buffer zones: (a) 100 m, (b) 200 m, (c) 500 m, and (d) 1000 m excluding phosphorus. The numbers within the plot represent the percentage of relative importance for each covariate.
**Figure S5:** Relative feature importance after applying the FAI threshold of 0.02 across different buffer zones: (a) 100 m, (b) 200 m, (c) 500 m, and (d) 1000 m excluding slope and elevation. The numbers within the plot represent the percentage of relative importance for each covariate.
**Figure S6:** (a) Total maximum bloom extent (MBE) area per region using thresholds of 0.02 and 0.05. Note that the area is the combination of 2 years areas. (b) Relative feature importance based on the FAI threshold of 0.05 at the 100 m buffer.
**Table S1:** Number of satellite images used for each lake by country for 2021 and 2022. Note that one image represents 1 month. If multiple images were available for a particular month, we mosaicked them to create a single image.

## Data Availability

The data that support the findings of this study are openly available: The Sentinel‐2 Level‐2 product used in this study was downloaded from https://dataspace.copernicus.eu/. The HydroLAKES database was obtained from https://www.hydrosheds.org/products/hydrolakes. The code for the random forest model is available at: https://github.com/ETHZ‐repositories/Eutrophication_SE.
