# Supplementary material for: Soil Erosion as a Driver of Eutrophication: An Analysis of European Lakes Using Sentinel‐2 Satellite Data
Source: Glob Chang Biol. 2025 Sep 12;31(9):e70494. doi: 10.1111/gcb.70494 (PMC12427167; doi:10.1111/gcb.70494)
Supplement: Supplementary file 1 — Data S1: Relationship between maximum bloom extent (MBE) and land use land cover. Figure S1: Relative importance of individual covariates: (a) flow accumulation, (b) phosphorus, (c) nitrogen, (d) temperature, (e) elevation, and (f) slope with respect to different buffer sizes. Figure S2: The relationship between phosphorus inflow concentration, water residence time, and trophic state. Figure from Vollenweider and Kerekes (1982). Figure S3: Relationship between eutrophication area/maximum bloom extent and the percentage of different land use/land cover (LULC) types. Panels (a, d, g, j) depict forest cover, (b, e, h, k) represent agricultural land, and (c, f, i, l) illustrate built‐up areas for France, Hungary, Germany, and Austria, respectively. Note the unit of eutrophication area is log10 ha. Figure S4: Relative feature importance after applying the FAI threshold of 0.02 across different buffer zones: (a) 100 m, (b) 200 m, (c) 500 m, and (d) 1000 m excluding phosphorus. The numbers within the plot represent the percentage of relative importance for each covariate. Figure S5: Relative feature importance after applying the FAI threshold of 0.02 across different buffer zones: (a) 100 m, (b) 200 m, (c) 500 m, and (d) 1000 m excluding slope and elevation. The numbers within the plot represent the percentage of relative importance for each covariate. Figure S6: (a) Total maximum bloom extent (MBE) area per region using thresholds of 0.02 and 0.05. Note that the area is the combination of 2 years areas. (b) Relative feature importance based on the FAI threshold of 0.05 at the 100 m buffer. Table S1: Number of satellite images used for each lake by country for 2021 and 2022. Note that one image represents 1 month. If multiple images were available for a particular month, we mosaicked them to create a single image. [file GCB-31-e70494-s001.docx]

**Supplementary of “Soil erosion as a driver of eutrophication: an analysis of European lakes using Sentinel-2 satellite data”**

**Surya Gupta^1^, Simon Scheper^1,2^, Pasquale Borrelli^1,3^, Panos Panagos^4^, Christine Alewell^1^**

1: Department of Environmental Sciences, University of Basel, Basel, 4056, Switzerland

2: Dr. Simon Scheper - Research | Consulting | Teaching, DE-29413, Dähre, Germany

3: Roma Tre University - Department of Science, Largo San Leonardo Murialdo 1, 00146 Roma, Italy

4: European Commission, Joint Research Centre (JRC), Ispra, Italy

**Data S1: Relationship between maximum bloom extent (MBE) and land use land cover**

The relationship between MBE and land use/land cover (LULC) was also examined across different buffer zones. For this analysis, the ESA LULC dataset (https://esa-worldcover.org/en) at 10 m resolution was used. First, 100 m buffer zones around each lake were overlaid with the LULC data to extract land cover information. Then, for each lake’s buffer, the percentage area of different land cover classes such as forest, agriculture (cropland and grassland), and built-up areas was calculated." Finally, these percentage values were visualized using box plots to assess the correlation between MBE and LULC, providing insights into how land cover influences MBE.

**Table S1:** Number of satellite images used for each lake by country for 2021 and 2022. Please note that one image represents one month. If multiple images were available for a particular month, we mosaicked them to create a single image.

|  | Number of lakes | | | | | |
| --- | --- | --- | --- | --- | --- | --- |
| No of images | UK | Poland | Hungary | Germany | France | Austria |
| 1-3 | 21 | -- | -- | -- | -- | 28 |
| 3-6 | 15 | -- | -- | 59 | 1 | 53 |
| 6-10 | 231 | 258 | 29 | 91 | 208 | -- |
| >10 | 49 | 207 | 44 | 116 | 101 | -- |

| 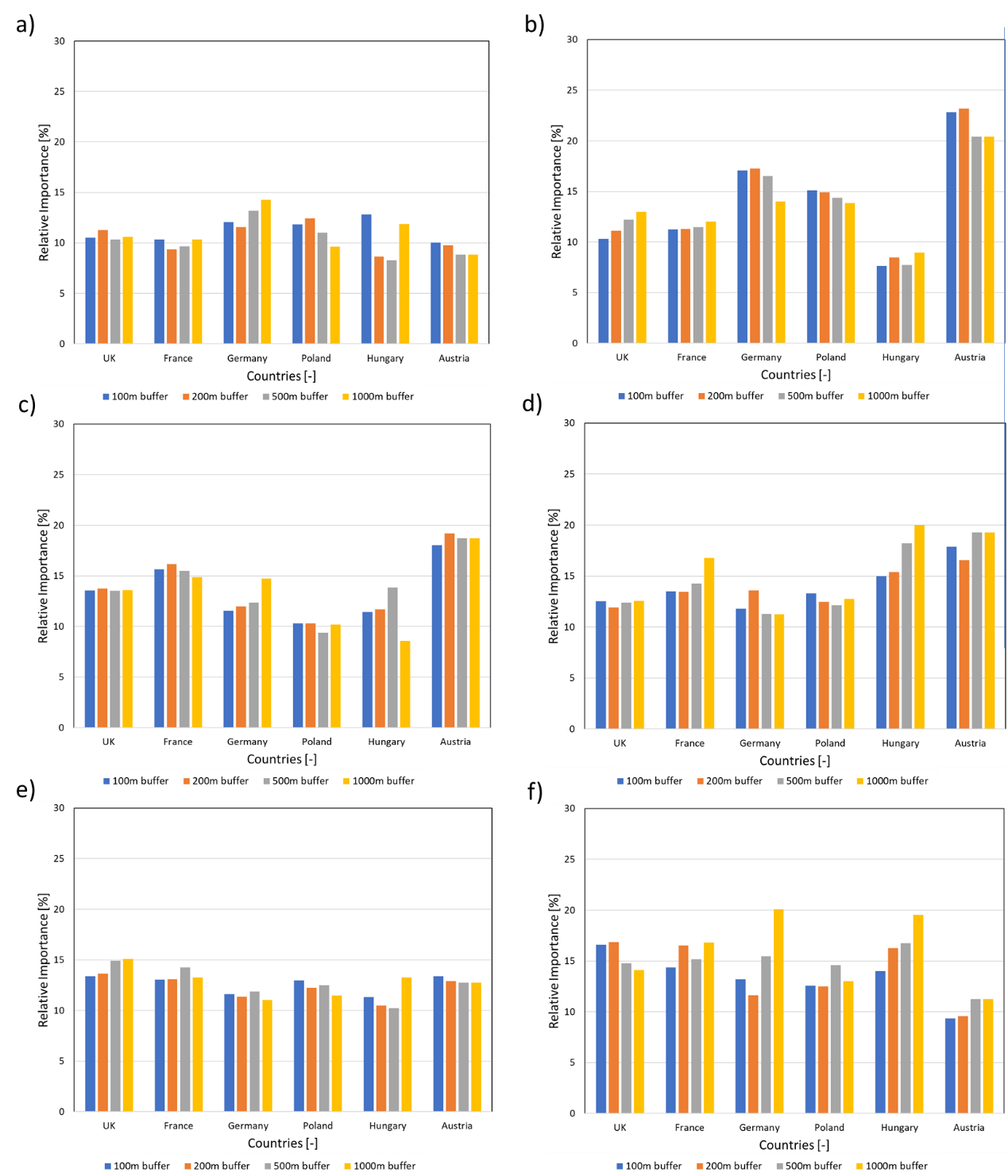 |
| --- |

**Figure S1:** Relative importance of individual covariates: a) flow accumulation, b) phosphorus, c) nitrogen, d) temperature, e) elevation, and f) slope with respect to different buffer sizes.

| **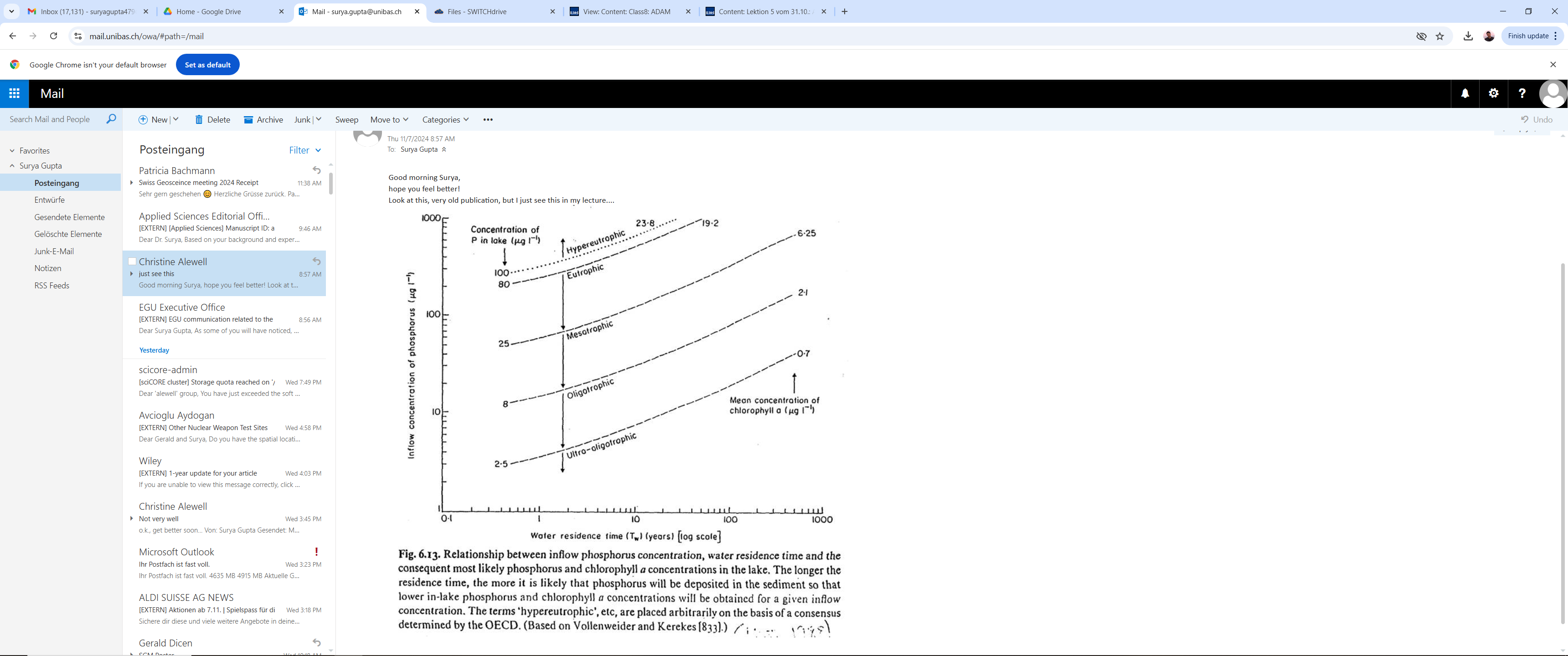** |
| --- |

**Figure S2:** The relationship between phosphorus inflow concentration, water residence time, and trophic state. Figure from Vollenweider and Kerekes (1982).

| **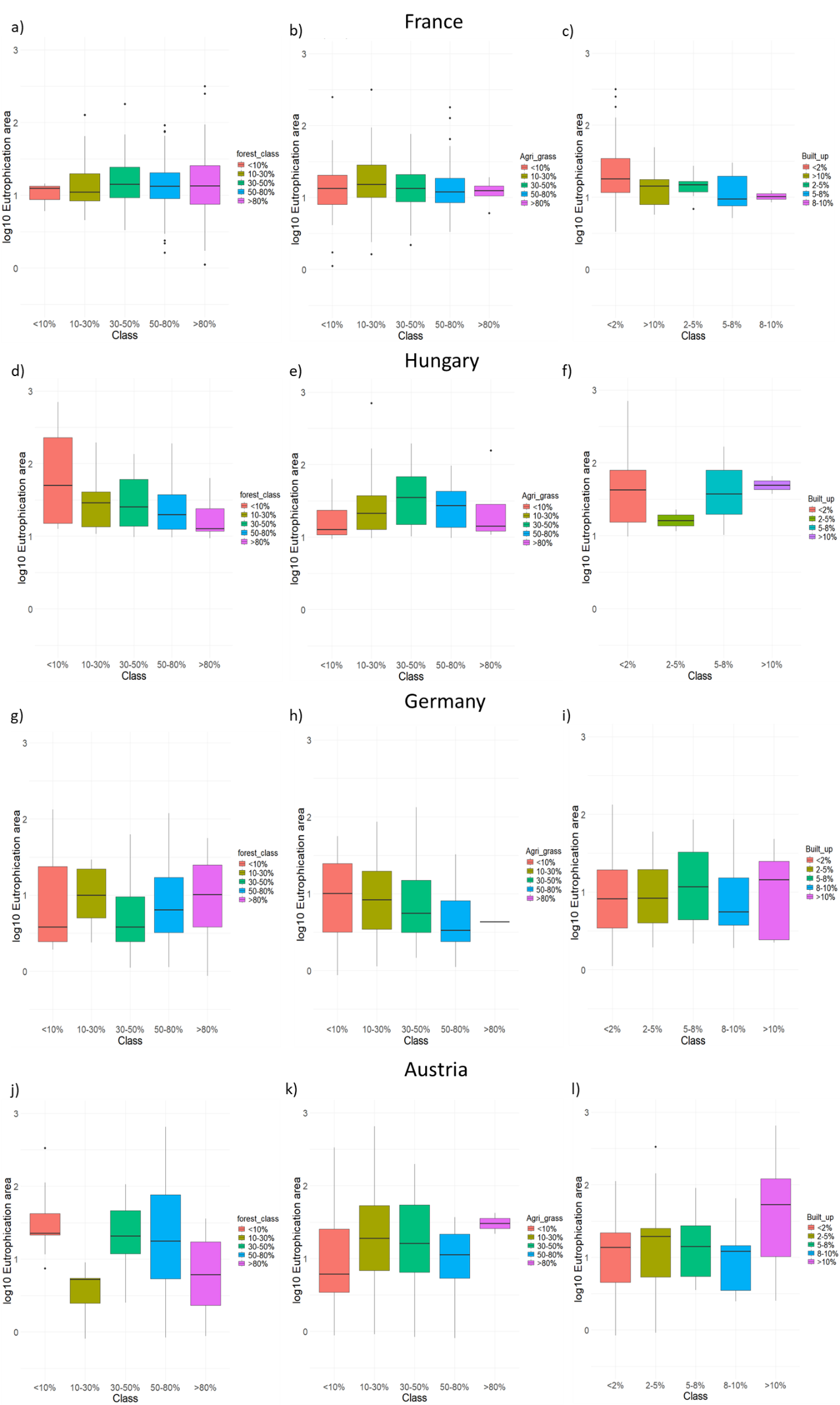** |
| --- |

**Figure S3:** Relationship between eutrophication area/maximum bloom extent and the percentage of different land use/land cover (LULC) types. Panels a), d), g) and j) depict forest cover, b), e), h), and k) represent agricultural land, and c), f), i), and l) illustrate built-up areas for France, Hungary, Germany and Austria, respectively. Note the unit of eutrophication area is log 10 ha.

| **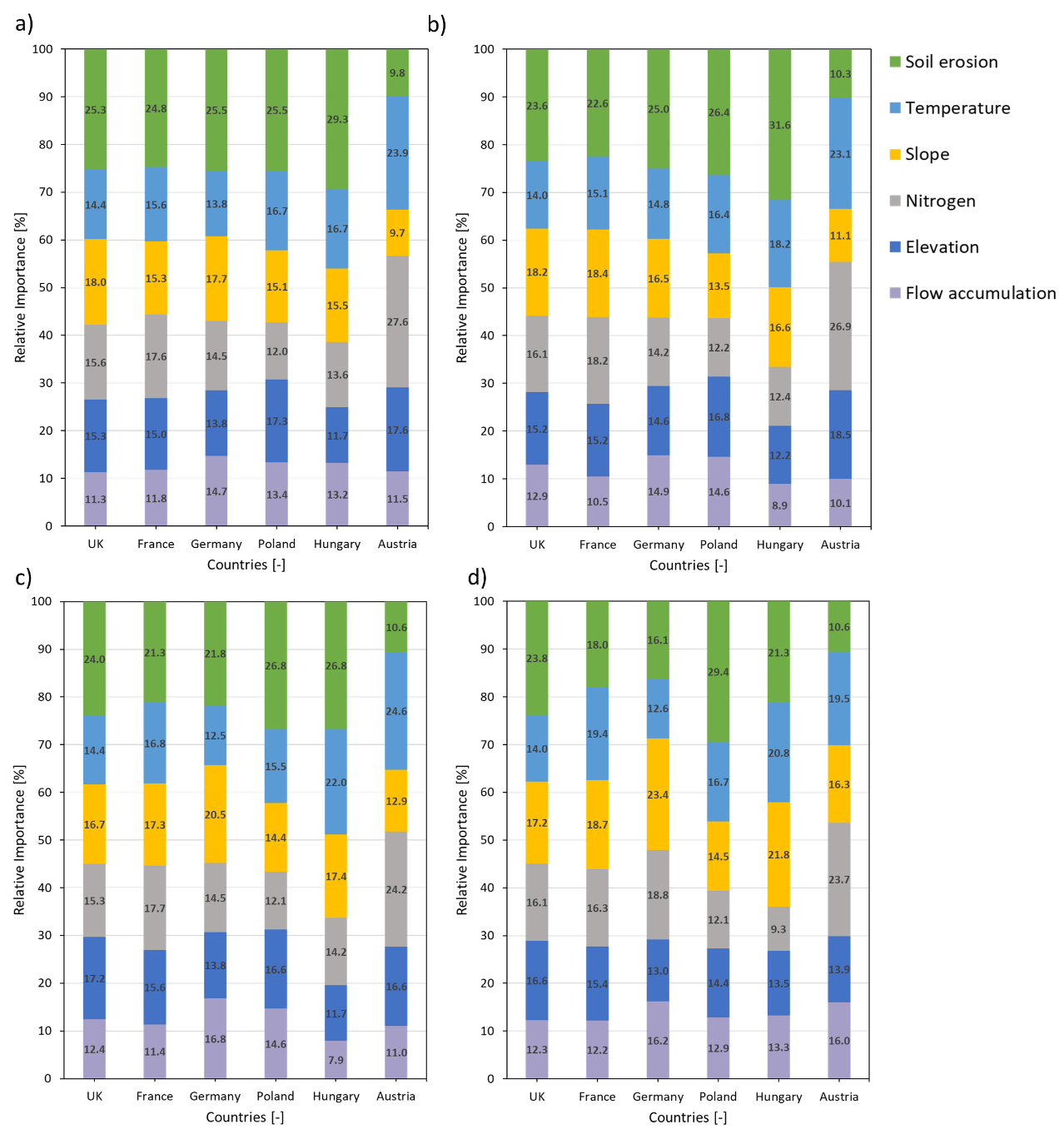** |
| --- |

**Figure S4:** Relative feature importance after applying the FAI threshold of 0.02 across different buffer zones: (a) 100 m, (b) 200 m, (c) 500 m, and (d) 1000 m excluding phosphorus. The numbers within the plot represent the percentage of relative importance for each covariate.

| 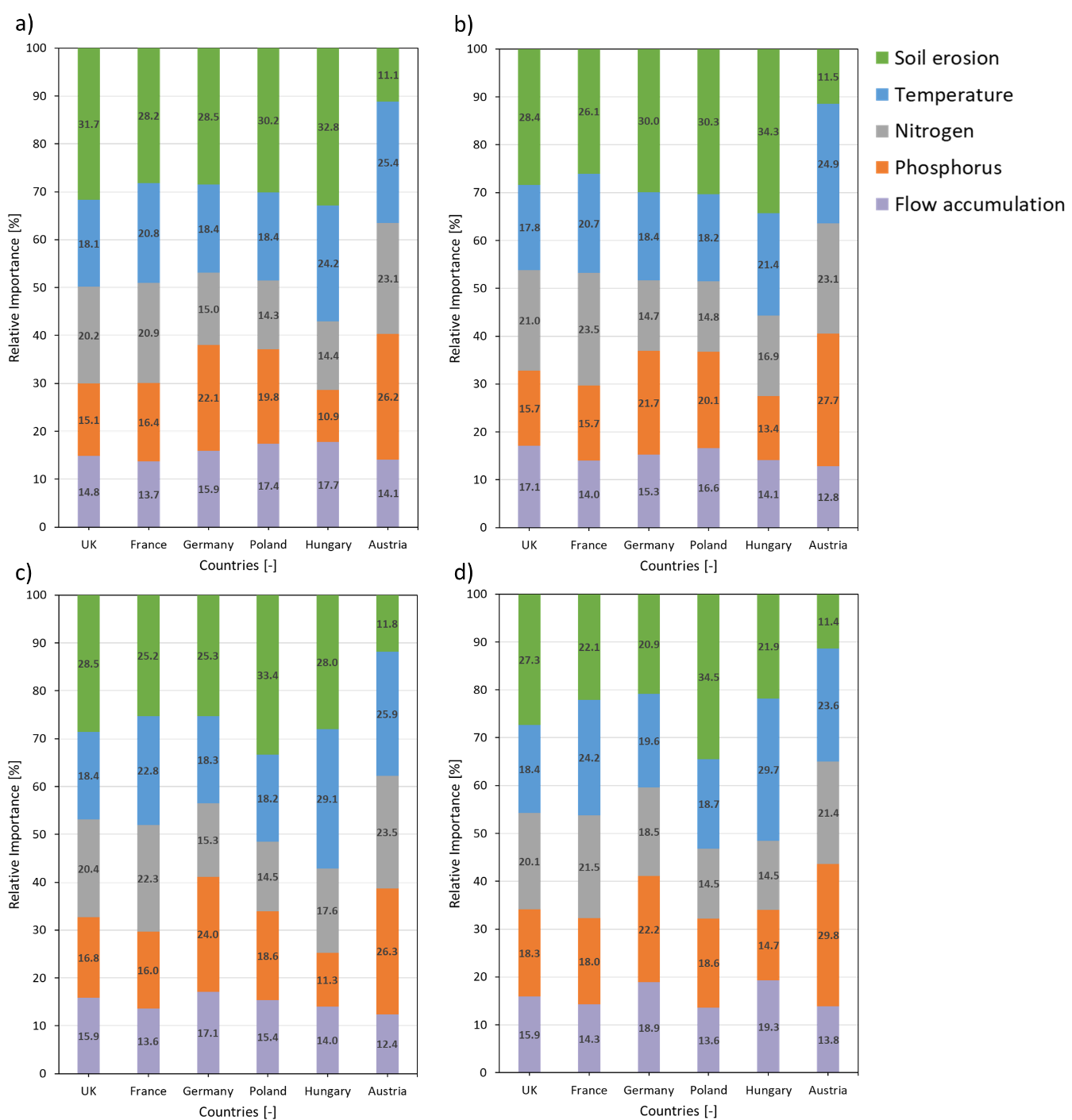 |
| --- |

**Figure S5:** Relative feature importance after applying the FAI threshold of 0.02 across different buffer zones: (a) 100 m, (b) 200 m, (c) 500 m, and (d) 1000 m excluding slope and elevation. The numbers within the plot represent the percentage of relative importance for each covariate.

| **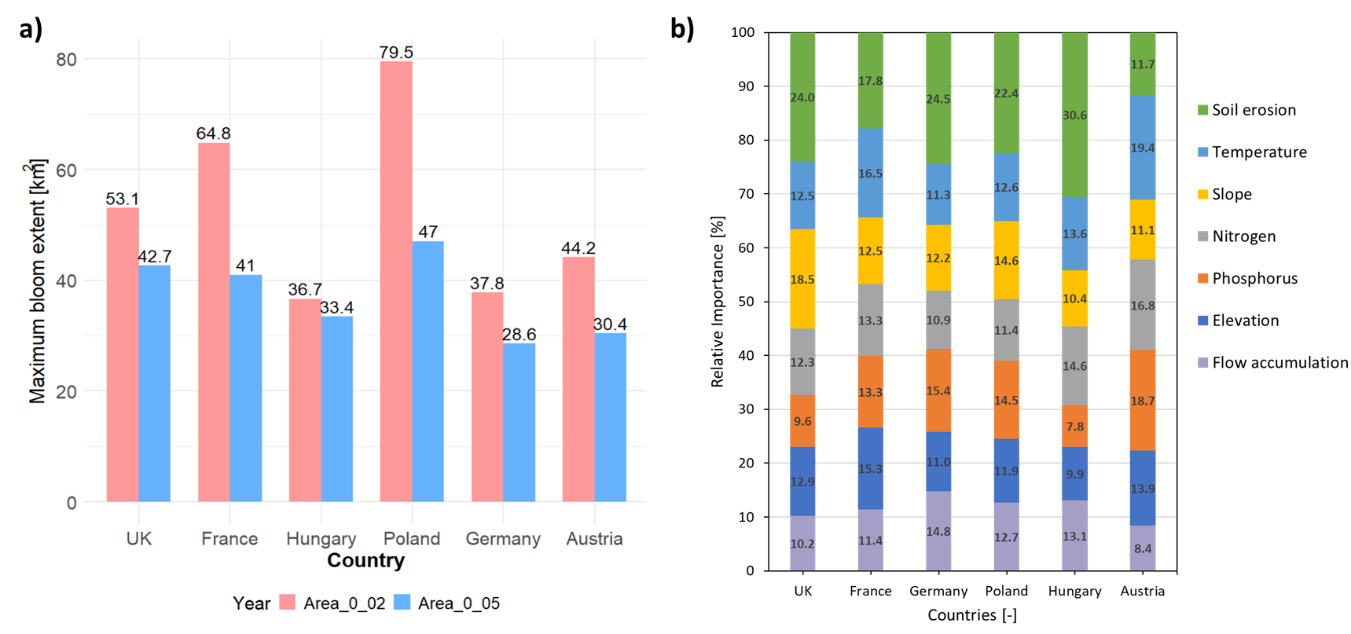** |  |
| --- | --- |

**Figure S6:** a) Total maximum bloom extent (MBE) area per region using thresholds of 0.02 and 0.05. Note that the area is the combination of two years areas. b) Relative feature importance based on the FAI threshold of 0.05 at the 100 m buffer.

**References**

Vollenweider, R. A., & Kerekes, J. (1982). Eutrophication of waters. Monitoring, assessment and control. Organization for Economic Co-Operation and Development (OECD), Paris, 156.
